# The Second Prescription

**Published:** 1888-08

**Authors:** J. T. Kent

**Affiliations:** Philadelphia


### THE SECOND PRESCRIPTION.

(I. H. A., June, 1888.)

J. T. KENT, M. D., PHILADELPHIA.

What is more beautiful to look upon than the bud during its hourly changes to the rose in all its bloom. This evolution has so often come to my mind when patiently awaiting the return of symptoms after the first prescription has exhausted its curative power. The return symptom-image unfolds the knowledge by which we know whether the first prescription was the specific or the palliative, *i. e.*, we may know whether the remedy was deep enough to cure all the deranged vital wrong or simply a superficial acting remedy, only able to sustain a temporary effect. The many things learned by the action of the first remedy determine the kind of demand made upon the physician for the second prescription.

Many problems come up to be solved that must be solved, or failure may follow.

How long shall I watch and wait? is a question frequently asked, but seldom answered. Is the remedy still acting? Is the vital reaction still affected by the impulse of the remedy? If the symptoms are returning, how long shall they be watched before it is necessary to act or give medicine? Is the disease acute or chronic? Why is the second prescription so much more difficult than the first? Why is it that so many patients are benefited when first going to the physician and thereafter derive no benefit?

I presume that most good prescribers will say, we have often acted too soon, but never waited too long. Many physicians fail because of not waiting, and yet the waiting must be governed by knowledge. Knowledge must be had, but where can it be obtained? To know that this waiting is right, is quite different from waiting without fixed purpose. This knowledge cannot be found where its existence is denied: it is not found with unbelievers and agnostics.

When the first prescription has been made, and the remedy has been similar enough to change the existing image, we have but to wait for results. The manner of change taking place in the totality of symptoms means everything, yet the manner of the return of the image, provided it has disappeared, means more.

*First.*—If aggravation of symptoms follow.

*Second.*—If amelioration of symptoms follow.

Aggravation of existing symptoms may come on with general improvement of the patient, which means well; but if aggravation of the symptoms is attended with decline of the patient, the cure is doubtful, and the case must be handled with extreme care, as it is seldom that such patients recover perfectly.

If amelioration follow the prescription, to whât does the amelioration apply? It may apply to the general state or but to the few symptoms. If the patient does not feel the elasticity of life returning, the improved symptoms are the facts upon which to doubt recovery. The knowledge that the disease is incurable is often obtained only in this way. In such cases, every remedy may palliate his sufferings, but cure does not come. The symptoms that are the expressions of the debility are there, and hence the totality of the symptoms is not removed.

After the curative impulse has entirely subsided, the symptoms will appear one by one, falling into place to arrange an image of the disease before the intelligent physician for the purpose of cure. If the first prescription has been continuously given, there has been little if any chance of a pure returning image of the disease, therefore, this image must be very unreliable. When the remedy has been fully exhausted, then, and only then, can we trust the symptoms constituting the picture.

If the first prescription was the simillimum, the symptoms will return, when they return, asking for the same remedy. Too often the remedy has been only similar enough to the superficial symptoms to change the totality, and the image comes back changed, therefore looking like the image of another remedy, which must always be regarded as a misfortune, by which the case is sometimes spoiled, and the hand of a master may fail to correct the wrong done. Whenever the symptoms return in the same image, calling for the same remedy, then it is that we have demonstrated that for a time, if the disease be chronic, we have but to commend the range of dynamics to cure this case. This rule is almost free from exceptions if the remedy is an antipsoric. What must the physician do who has not the knowledge of dynamic medicines? He must sometimes see sick images come back without change of symptoms, though I believe it is seldom. The symptoms may call for Phosphorus as strongly as when he began, and Phosphorus$^{6x}$ has served and no longer cures. What can he do but change his remedy? Can it be possible that man can be so ignorant of how to cure as to give a drug that is not indicated because the one that is indicated does not cure? These ignorant mortals condemn the system of Homœopathy and feel that they have performed their duty to the sick, forgetting that ignorance was the culprit. I have observed in cases where a low potency had been administered in frequently repeated doses that some time must elapse before a perfect action will follow the higher potency; but where the dose had not been repeated after its action was first observed, the new and higher potency will act promptly.

When the symptoms come back after prudent waiting, unchanged, the selection was correct, and if the same potency fail to act, a higher one will generally do so quite promptly, as did the lower one at first. When the picture comes back changed only by the absence of some one or more symptoms, and no new symptoms, the remedy should never be changed until a still higher potency has been fully tested, as no harm can come to a case from giving a single dose of a medicine that has exhausted its curative powers; it is even negligence not to do just this thing.

When the demonstration is clear that the present remedy has done all it is capable of doing, and this demonstration cannot be made until much higher potencies than usually made have been tried, then the time is present for the next prescription. To change to the next remedy becomes a ponderous problem, and what shall it be? The last appearing symptom shall be the guide to the next remedy. This is so whenever the image has been permitted to settle by watching and waiting for the shaping of the returning symptom-picture. Long have I waited after exhausting the power of a remedy, while observing a few of the old symptoms returning, finally a new symptom appears. This latest symptom will appear in the anamnesis as best related to some medicine having it as a characteristic which will most likely have all the rest of the symptoms. It is not supposed that this latter appearing symptom is an old symptom on its way to final departure; for so long as old symptoms come back and go, it is granted that no medicine is to be thought of.

It is an error to think of a medicine when a symptom-image is changing; the physician must wait for permanency or firmness in the relations of the image before making a prescription. Some say, "I must give the patient medicine or he will go and get some one else." I have only to say that it were better had all sick folks gone somewhere else, for these doctors seldom cure but often complicate the sickness.

The acute expressions of a chronic disease have a different management from the acute disease, *per se:* a child suffers from bronchitis every change of the weather, and may grow worse and worse if treated with the remedy for the acute symptoms. The miasm that predisposes the child to recurrent attacks must be considered. One recently under my care had received Antimonium tart., Calcarea, Sulphur, Lycopodium, etc., in such indiscriminate confusion that the child was not cured. The waiting on Sac. lac. through several attacks permitted the drugs to pass off and the true image of the sickness was permitted to express itself through several of the exacerbations taken as a whole. When Western ague is complicated with a miasm, a single paroxysm does not fully express the totality, but several must be grouped and the true image will be discovered. If the acute disease be uncomplicated with a miasm, the indicated remedy will wipe it out "*cito, tuto et jucunde.*"

All things oppose haste in prescribing. In very grave diseases haste is the common error, more frequently with the second prescription than the first. Many doctors suppose that a diphtheria demands an immediate medicine because "something must be done." This is an error; many a life has been saved by waiting and waiting. For example: A little girl was suffering from a severe attack of diphtheria, and mother had treated it four days with Mercurius$^{3x}$ and Kali bich.$^{3x}$ in alternation. She was poor, and therefore I did not refuse to take the case, which was then in a very bad state; nose, mouth, and larynx full of exudation. After a long study, the child received Lycopodium$^{cm}$ (F.), one dose dry, which cleared out all the exudation from nose and fauces, but did not touch the larynx. I dare not tell you how long I watched that child before I saw an indication for the second remedy, which it would have never needed had the Lycopodium been given when the child first took sick. I waited until the poor child was threatening dissolution, when I saw a little tough, yellow mucus in the mouth; Kali bich.$^{cm}$, one dose, cleared the larynx in one day and there was no further indication necessary.

The first prescription is made with the entire image of the sickness formed. People usually send for the doctor after there can be no doubt of the sickness to be treated; the doctor watches the improvement of the patient and the corresponding disappearance of the symptoms under the first prescription, and when the case comes to a standstill he is uneasy, and with increasing fidgetyness he awaits the coming indications for the next dose of medicine. Often he does not wait, and hence the reports of lingering sicknesses in our medical journals. This fidgetyness, which comes from lack of knowledge, unfits the physician as an observer and judge of symptoms; hence we see the doctor usually failing to cure his own children. He cannot wait and reason clearly over the returning symptoms. The first prescription may have been correct, but the second prescription is dangerous to make in a hurry.

While watching the prescriptions of beginners, I have observed very often the proper results of the first prescription. The patient has improved for a time, then ceased to respond to any remedy. Close investigation generally reveals the fact that this patient improved after the first dose of medicine, that the symptoms changed slightly without new symptoms, and the new "photo" seemed to call for some other remedy, when, of course, the remedy was changed and trouble began; constant changing of remedies followed until all the antipsorics in the *Chronic Diseases* had been given on flitting symptom-images and the patient is yet sick. This is the common experience of young Hahnemannians trying to find the right way. Some of experience make lesser blunders and some make few, but how many have made none? All of these blunders I have made, as I had no teacher, until I blundered upon the works of the great master.

The third great mistake (*Chronic Diseases*) which the homœ-

opathic physician cannot too carefully avoid in the treatment of chronic diseases is the too hasty repetition of the dose. The three precautions of the master found in the *Chronic Diseases* should be printed and posted in every physician's office and committed to memory. The third precaution relates to the second prescription.

The first prescription may not have been a well-chosen medicine, and then it becomes necessary to make a second effort. As time brings about the re-examination of the patient, new facts are brought out in relation to the image of the sickness that show that the first medicine had not been suitable; perhaps several weeks have passed and the re-examination shows no change in the symptoms. Shall I compare anew all the facts in the case to reassure myself of the correctness of the first prescription, or shall I wait longer? Yes, to the former, of course, and if the remedy still is the most similar of all to the symptoms, wait and watch and study the patient for a new light on his feelings that he has become so accustomed to that he has not observed. Commonly the new study of the case will reveal the reason why the first prescription has not cured; it was not appropriate. If it still appear to be the most similar remedy the question comes up, "How long shall I wait?" Also the change of potency may be considered. It is the practice of some to go higher, but the first dose may have been very high and then the previous question is to be considered, "How long shall I wait?"

At this point it should be duly appreciated that the length of time is not so important as being on the safe side, and "wait" is the only safe thing to do. But it may have been many days; but that matters not, wait longer. The finest curative action I ever observed was begun sixty days after the administration of the single dose. The curative action may begin as late as a long-acting drug can produce symptoms on the healthy body. This guide has never been thought of by our writers, but is well to be considered. Why not? It is the practice for some to go lower if a high potency has failed. This method has but few recorded successes, but should not be ignored.

The question next to be considered is the giving of a dose of medicine in water and in divided doses. This has at times seemed to have favor over the single dose dry. This is open for discussion, requiring testimony of the many, not few, to give it weight. The best of reports are made of both methods, and both are in harmony with correct practice.

The next important step to be considered is when the first prescription has acted improperly or without curative results. Then it becomes necessary to consider a second prescription. The first prescription sometimes changes the symptoms that are harmless and painless into symptoms that are dangerous and painful. If a rheumatism of the knee goes to the heart under a remedy prescribed for the one symptom, the remedy has done harm; it is an unfortunate prescription and must be antidoted. If its antidote is not known the new symptoms must be prescribed for. Whenever symptoms are changed from surface to centre, the medicine must have an antidote. In incurable diseases when a remedy has set up destructive symptoms, an antidote must be considered.

If the medicine changes the general symptom-image and the general state of the patient is growing worse, the question then comes up, Was the prescription only similar to a part of the image, or, is the disease incurable? Knowledge of disease may settle this question. If the disease is incurable, the action of the remedy was not expected to do more than to change the sufferings into peaceful symptoms, and a second prescription is to be considered only when new sufferings demand a remedy. But suppose such a change of suffering comes after the first prescription, and the disease is undoubtedly curable, then the conclusion must be that the first prescription was not the true specific, and that the true image has not been seen. The second prescription is then to be considered, but hastiness may spoil all, as the first prescription has nearly done. Wait until the old image has fully returned is all there is to do. A prescription of the remedy that might have cured would now be useless in a chronic case. It is hazardous practice to follow up rapidly all the changing symptoms in any sickness with remedies that simply for the moment seem similar to the symptoms present. The observing physican will know by the symptoms and their directions whether the patient is growing better or worse, even though he appeared to the contrary to himself and friends. The complaints of patient or friends constitute no ground for a second prescription. The greatest sufferings may intervene in the change of symptoms in progress of permanent recovery, and if such symptoms are disturbed by a new prescription or palliated by inappropriate medicine, the patient may never be cured.

The object of the first prescription is to arrange the vital current or motion in a direction favorable to equilibrium, and when this is attained it must not be disturbed by a new interference. Ignorance in this sphere has cost millions of lives: when will the medical world be willing to learn these principles so well that they can cure speedily, gently, and permanently? There can be no fixed time for making the second prescription ; it may be many months. The second prescription must be one that has a friendly relation to the last one or the preceding. No intelligent prescription can be made without knowing the last remedy. Concordances in Bœnninghausen must not be ignored. The new remedy should sustain a complementary relation to the former.

In managing a chronic sickness the remedy that conforms to an acute experience of the illness is worth knowing, as very often its chronic may be just the one that conforms to the symptoms. Calcarea is the natural chronic of Belladonna and Rhus. Natrum mur. sustains the same relation to Apis and Ignatia ; Silicea to Pulsatilla ; Sulphur to Aconite. The fact that Pulsatilla has been of great service in a given case and finally cures no longer, but the symptoms now point to Silicea, the latter will be given with confidence as its complementary relation has long been established. While, on the other hand, Causticum and Phosphorus do not like to work after each other, nor will Apis do well after Rhus.

How physicians can make the second prescription without regard to the experience of nearly a century, is more than man can know. These things are not written to instruct men of experience in the right way, but the young men who have asked so often for the above notes of our present practice. I am told almost daily that this kind of practice is splitting hairs, but I am more and more convinced of the necessity of obeying every injunction.

You should have no confidence in the experience of men who do not write out faithfully all the symptoms of patients treated, and note carefully the remedy and how given : especially is this necessary in patients likely to need a second prescription. The physician who has in his case-book the notes of every illness of his patients, has wonderful hold of any community. He has the old symptoms and the remedies noted that cured, and he can make indirect inquiry after all the old symptoms long ago removed. The pleasure is not small found in consulting such a note-book. Experience soon leads the close prescriber to note all the peculiar symptoms and to omit the nondescript wanderings indulged in by all sick people ; however, it is important to be correct in judgment.

Many physicians make a correct first prescription and the patient does well and cheers up for awhile, but finally the test is made for the second, and then all is lost. Homœopathy is nothing if not true, and, if true, the greatest accuracy of detail and method should be followed. It is fortunate that the physicians who repeat while the remedy is acting are such poor prescribers, or their death list would be enormous.

A prominent writer has boasted that he could and had repeated high attenuations without effect. There can be no stronger confession than this of ignorance as to the knowledge of selecting remedies. Such men do not and cannot see their own lack of knowledge, or they would know why the statement is only a self-condemnation. Can it be that one physician who reads this will be urged to be more accurate in his habit when making the second prescription; if not, this effort is lost.

I have no hope of reaching such men as have only the desire to be scientific. Hahnemann never thought of establishing a science of medicine, but everywhere calls it the "Art of Healing." The sooner it is settled that men who are everlastingly seeking to be scientific and demonstrating this scientificity by chemistry, pathology, and the microscope are not homœopaths, neither indeed can be, the better it will be for the followers of law and truth.

The man who works for the mighty dollar cannot be reached by this paper. I am well aware that it will act upon him as do the raindrops upon the well-oiled fowl; neither can the vital spark look to him for protection. Yet a few will find their own efforts and experiences verified in this paper, and a few will profit by the recorded rules that have grown out of following law.

